# A meta-analysis of death anxiety in people living with or beyond cancer: the important role of culture

**DOI:** 10.1007/s11764-025-01856-8

**Published:** 2025-07-04

**Authors:** Audrey Bennett, Louise Sharpe, Jack B. Boyse

**Affiliations:** School of Psychology, The University of Sydney, Sydney, NSW Australia

**Keywords:** Cancer, Oncology, Psycho-oncology, Psychosocial, Fear of cancer recurrence, Culture, Thanatophobia

## Abstract

**Purpose:**

This review explored associations between death anxiety and a range of psychosocial outcomes, such as fear of cancer recurrence (FCR), depression, anxiety, and quality of life, in people living with or beyond cancer. We also investigated culture (individualistic versus collectivist) as a moderator of the relationship between death anxiety and psychosocial outcomes.

**Methods:**

We included studies that measured death anxiety and one or more psychosocial outcomes in people with a history of cancer. Forty-one studies were included and meta-analysed to identify the strength of relationships between death anxiety and psychosocial outcomes. Cultural orientation was investigated as a moderator of these relationships. The quality of included studies was appraised.

**Results:**

Death anxiety was strongly associated with all psychosocial outcomes, including increased FCR, depression, anxiety, and poorer quality of life, self-esteem, and sense of meaning. Younger people and those currently in active treatment had higher levels of death anxiety. The relationship between death anxiety and depression was stronger in collectivist than individualistic cultures. Collectivist versus individualistic cultures differed in their death anxiety depending on the outcome measure used.

**Conclusions:**

Death anxiety is an important construct for people living with or beyond cancer, and this review confirms that different cultures may be concerned about different aspects of death, which warrants further empirical research.

**Implications for cancer survivors:**

These findings highlight the importance of providing targeted and culturally sensitive psychological interventions for death anxiety in those living beyond cancer.

**Supplementary Information:**

The online version contains supplementary material available at https://doi.org/10.1007/s11764-025-01856-8.

Death anxiety has been defined as the fear, panic, and emotional distress associated with one’s awareness of mortality [1]. For those living with life-threatening diseases such as cancer, the fear of death is particularly salient. Advances in early detection and treatment of cancer have seen an increase in survival rates for those diagnosed with cancer [2]. In Australia, the rate of people surviving at least 5 years after a diagnosis has increased from 51 to 70% over the last 30 years [3]. This increase means people are living longer after receiving treatment either with no evidence of disease or with advanced disease.

Yet, amongst people in the community, cancer is still commonly associated with death [4]. For example, ‘death’ is the first thought of most people after hearing the word ‘cancer’ [5]. As such, a diagnosis of cancer is understandably a trigger for existential issues, including death anxiety [6]. Nevertheless, elevated and persistent levels of death anxiety have been found to prevent effective end-of-life planning and are associated with poorer quality of life (QoL) [7]. Conversely, low levels of death anxiety are associated with better mental health outcomes and higher QoL [8].

The links between poor mental health and death anxiety in people with cancer are hardly surprising, since research outside the cancer field has implicated death anxiety as a transdiagnostic construct underpinning several mental health conditions [9]. Despite the saliency of death anxiety in the cancer context, the literature has been slow to fully investigate the role of death anxiety and its relationship with key psychosocial issues, such as FCR [10]. However, in the past few years, research has proliferated, and four meta-analyses have been conducted that examine aspects of death anxiety in a cancer context.

Two meta-analyses investigated the severity of death anxiety in cancer patients. Soleimani et al. [11] identified 15 studies and overall found moderate levels of death anxiety amongst those living after a diagnosis of cancer, with the majority of studies using the Death Anxiety Scale. Death anxiety was found to be higher in studies including a higher proportion of breast cancer patients, women, or married participants. A similar result was identified by Shakeri et al. [12] who identified 23 studies of death anxiety, all using the Death Anxiety Scale, specifically in Iranian people with cancer. They also found moderate to high levels of death anxiety in the Iranian cohort and found that death anxiety was higher in samples with a larger proportion of breast cancer survivors. Importantly, neither of the two reviews assessed whether there was an association between death anxiety and psychosocial outcomes in cancer patients.

The third meta-analysis investigated treatments for fears of death in advanced cancer patients [13]. They identified five randomised controlled trials, though these trials had several serious methodological limitations. Overall, the study found weak evidence that interventions focusing on spiritual well-being and meaning-making were beneficial in reducing death anxiety. However, the efficacy of these interventions for death anxiety could not be confirmed due to the lack of rigorous analysis and only one study [14] using a validated measure of death anxiety.

Most recently, Li et al. [15] published a meta-analysis of demographic and psychosocial correlates of death anxiety in cancer patients. Across 52 studies, they found that death anxiety was associated with being female (*z* = 0.24) and a younger age (*z* = 0.17). Larger correlations were found between psychosocial variables and death anxiety than between demographic variables and death anxiety. Namely, anxiety, depression, and fear of cancer recurrence (FCR) were all found to be strongly associated (effect sizes ranged from *z* = 0.41 to 0.66). Other variables including self-esteem, QoL, and sense of meaning were moderately and negatively associated with death anxiety (effect sizes ranged from *z* =  − 24 to − 0.41).

These four reviews have advanced our knowledge such that we know (1) death anxiety is elevated in people with cancer; (2) is worse in those who are younger, married or female and who have breast cancer and is associated with higher symptom burden and poorer psychosocial outcomes; and (3) there are some therapies with the potential to reduce death anxiety for people with advanced disease. However, there are gaps in our knowledge. Li et al. [15] found high heterogeneity across studies, but they did not investigate moderators of the relationship between death anxiety and psychosocial outcomes. One factor known to influence conceptualisations about death is culture [16]. We know that death anxiety is at least moderately elevated in people with a history of cancer across cultures, but death anxiety is higher in some regions (e.g. Asia) than others (e.g., USA) [11]. However, no previous meta-analysis has specifically investigated culture and death anxiety.

Conceptions of death and dying vary widely among different cultural and religious groups [16]. For instance, Día de los Muertos (Day of the Dead) in Mexico is a day of great celebration, where symbols of death are embraced as it is believed the spirits of the dead return to those who are living [17]. As death is so readily associated with celebration in Mexican culture, one might expect individuals to feel lower levels of fear towards their own death. In contrast, Western cultures tend to cremate or bury their dead, typically followed by solemn funerals and limited ongoing rituals. Therefore, it may be expected that the experience of death anxiety may be higher in cultures where solemn and funereal attitudes to death prevail. One aspect of culture, which Walter [18] has argued impacts societies and their management of death, is the degree to which the culture adheres to collectivist versus individualistic cultural norms.

Current research into the impact of individualistic versus collectivist cultures is sparse and conflicting [19]. For example, in the meta-analysis of death anxiety in people with a history of cancer, levels of death anxiety were estimated to be moderate overall but higher in Asian countries than in Western countries [11]. Similarly, in the meta-analysis focused solely on Iranian studies, levels of death anxiety were in the moderate to severe range, indicating a potentially heightened level of death anxiety [12]. These results are consistent with some of the broader research suggesting that eastern-collectivist cultures may experience higher death anxiety compared to western-individualist cultures [20].

These results, which suggest that higher levels of death anxiety in eastern-collectivist cultures are at odds with some accounts of cross-cultural differences in death anxiety [21]. Eastern-collectivist cultures commonly value interdependence, where an individual’s self-concept is predicated on their connection to their cultural in-group. Theorists have predicted that this interdependence and connection would buffer against death anxiety because of increased social support, and part of their identity exists in their group rather than through individual legacy. Further, many collectivist cultures are more spiritual and therefore believe in an afterlife. For example, Kashima et al. [21] found that those from collectivist cultures had lower levels of both collective and personal mortality fears than those from individualistic cultures. It may be that these mixed findings in the literature arise because different cultures may be concerned about different aspects of death. That is, individuals from collectivist cultures may have additional concerns about the death of others with whom they are more highly interdependent, whereas those from individualistic cultures may be more concerned about their own death [19]. In that case, one crucial issue in determining the nature of relationships may be how death anxiety is measured. However, the impact of collectivist versus individualistic cultures on levels of death anxiety, and the relationships between death anxiety and mental health outcomes have not been directly compared in the cancer literature. The present study aims to fill that gap, as well as investigating other factors that might moderate these relationships.

## Aims and hypotheses

The current meta-analysis will quantify relationships between death anxiety and psychosocial outcomes with a view to identifying moderators of these relationships. Specifically, we will examine whether those individuals from collectivist-eastern cultures and individualist-western cultures have different levels of death anxiety; and/or whether the relationships between death anxiety and psychosocial outcomes differ between different cultures. Furthermore, we will also explore the association between death anxiety and psychosocial outcomes and whether these associations differ as a function of cancer status.

## Method

### Search strategy

The protocol for this systematic review was preregistered with PROSPERO [CRD: 42023440788]. The search aimed to identify peer-reviewed quantitative studies which focused on death anxiety and psychosocial outcomes in people who have been diagnosed with cancer. The search was conducted on 30th June 2023. Studies were identified through a search of PsycINFO, PubMed, EMBASE, and CINAHL. The search was restricted to English studies published after January 1999. Key search terms were:

- (‘cancer’ or ‘neoplasm’ or ‘carcinoma’)
- (‘death anxiety’ or ‘fear of death’ or ‘mortality salience’)

Reference lists of included papers were also searched. Additionally, authors of studies which were identified in our search but could not be accessed were contacted to provide relevant information. All abstracts were independently reviewed by two of the authors (AB, LS) and full-text screenings were performed by two of three authors (AB, JB and LS). Differences in ratings were resolved through consensus.

### Inclusion criteria

Studies were eligible to be included in this systematic review if they (1) were peer reviewed, (2) were published in English (for pragmatic reasons), (3) were published from 1999 to June 2023, (4) featured a sample of cancer patients or survivors, (5) included a measure of death anxiety, and (6) included an analysis of how death anxiety relates to any psychosocial outcome (e.g., the participants’ mental health, health behaviours, fear of cancer recurrence/progression, QoL, and health care utilisation). We limited the publication years as cancer treatments and prognosis have changed dramatically in the past 20 years. There were no restrictions on other demographic characteristics, including age, gender, types of cancer, or stage of cancer.

### Data extraction

A data extraction template was created specifically for the present study. All coding was completed by the first author and JB independently. Disagreements were resolved via consensus. Data extracted from the articles at the study level were the year of publication, name and contact details of the lead author, journal name, country of research, funding sources, and study design. We extracted the following demographics: average age of the sample and proportion female. We also extracted cancer characteristics such as type of cancer, cancer stage, proportion of the sample with active disease, and treatment approach (e.g., treatment, palliation or survivorship). We also extracted data on the measures used to assess death anxiety and psychosocial outcomes, as well as statistical information such as mean, standard deviation, and sample size. Our primary outcomes of interest were the correlations between death anxiety and psychosocial outcome measures: depression, anxiety, FCR, QoL,self-esteem, demoralisation, and sense of meaning in life.

### Quality assessment

Risk of bias was assessed using the Joanna Briggs Institute Critical Appraisal Checklist [22] (Joanna Briggs Institute, 2020). For each item on the checklist, studies were scored as (1) meets criteria; or (0) does not meet criteria or (?) unclear. The first author and JB independently reviewed each study included in the review. The consistency between the authors’ ratings was excellent (*k* = 0.80). Any discrepancies were discussed and resolved through consensus. See Appendix A for the quality assessment template.

### Statistical analysis

Analyses were conducted with the Comprehensive Meta-Analysis program [23], using a random-effects model because we expected considerable heterogeneity between the included studies. Effect sizes were determined using Pearson’s *r* correlations between death anxiety and psychosocial outcomes and were considered small, medium, and large associations at 0.1, 0.3, and 0.5, respectively. Heterogeneity was assessed using the *Q* and *I*^2^ statistic. The risk of publication bias was assessed using funnel plots and fail-safe *N*. We provided a meta-analytic estimate of the effect where there were at least three eligible studies that could be included.

## Results

**Fig. 1:**
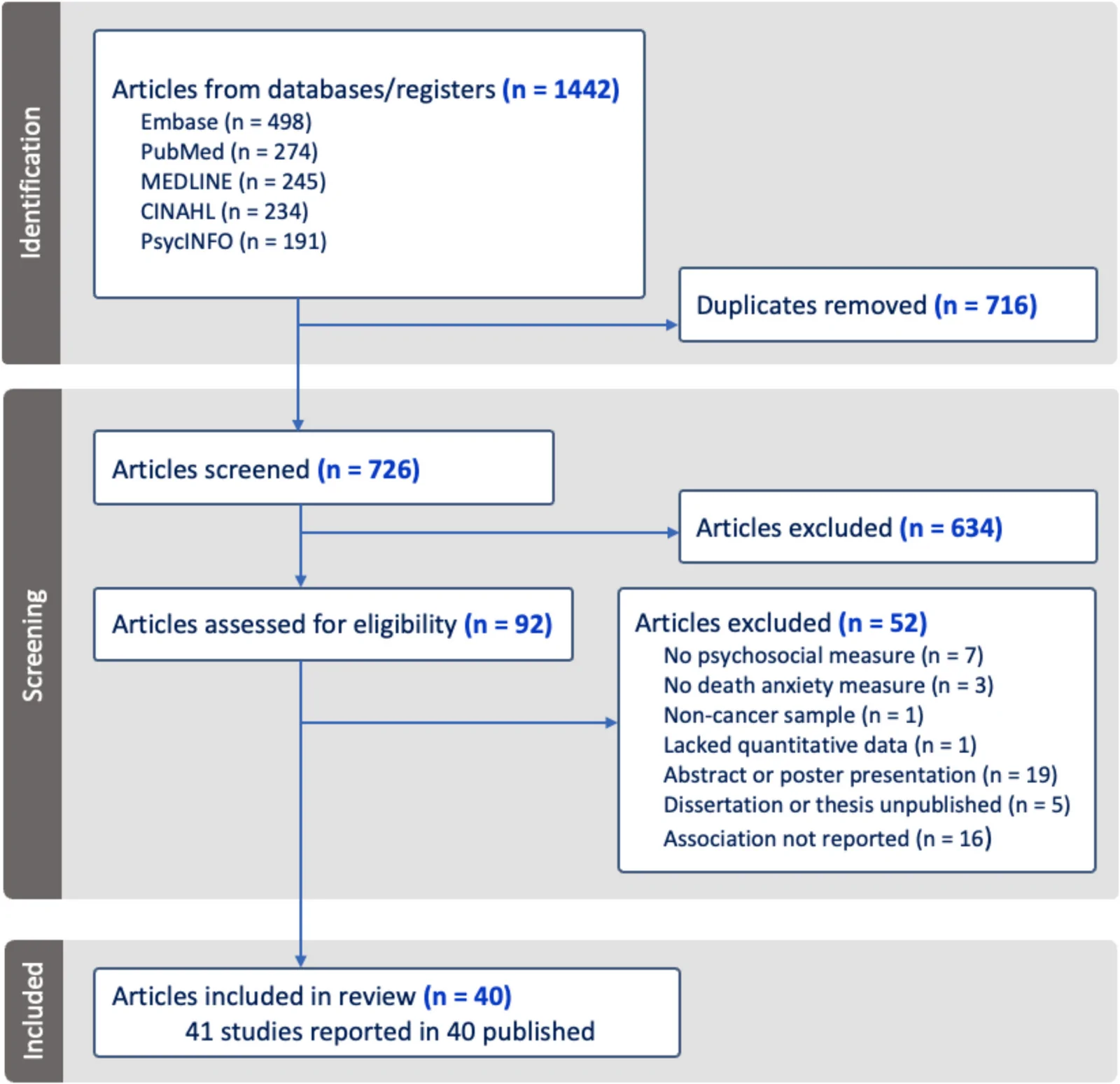
PRISMA flowchart of study selection process

In total, 1442 studies were retrieved from the database search, with 726 remaining after duplicates were removed. After title and abstract screening, the full texts of 89 studies were retrieved and resulted in 40 articles reporting 41 independent studies that were eligible for inclusion in the current review. The study selection process is illustrated in Fig. 1.

### Study characteristics

Studies were published between 2010 and 2023. All studies were cross-sectional, except for one longitudinal study; however, we used only the baseline data for the meta-analysis [24]^.^ The sample sizes of the 41 included studies ranged from 33 to 586, with a total of 7323 people living with or beyond cancer included. Table 1 shows the demographic and cancer characteristics of the included studies.

**Table 1:** Study characteristics and quality score

Author (year)	Country	Study design	Sample size	Cancer type	Cancer stage	Setting	Treatment approach	Average duration of illness	DA measure	Quality score (/8)
Abbas et al. [25]	Pakistan	Cross-sectional	200	Mixed	Mixed	Out-patient	Treatment	NS	DAS	5
Afrashteh and Masoumi [26]	Iran	Cross-sectional	210	Breast	Mixed	Not specified	Survivorship	NS	DAS	5
An et al. [27]	Canada	Cross-sectional	307	Mixed	Advanced	Out-patient	Treatment	NS	DADDS	8
Berlin and von Blackenburg [28]	Germany	Cross-sectional	121	Mixed	Mixed	Not specified	Mixed	NS	DADDS	5
Berlin et al. [29]	Germany	Cross-sectional	84	Mixed	Mixed	Not specified	Mixed	NS	DADDS	4
Bibi and Khalid [30]	Pakistan	Cross-sectional	80	Breast	Mixed	Both	Treatment	NS	DAS (Likert)	7
Bovero et al. [31]	Italy	Cross-sectional	200	Mixed	Advanced	In-patient	Palliation	NS	DADDS	6
Braun et al. [32]	USA	Cross-sectional	80	Mixed	Mixed	Not specified	Treatment	4.6 years	DAS	7
Coutts-Bain et al. [33]	Australia	Cross-sectional	176	Gynaecological	Mixed	Out-patient	Mixed	NS	DAQ	7
Curran et al. [34]	Australia	Cross-sectional	211	Mixed	Mixed	Out-patient	Mixed	5.9 years	DAQ	6
Eggen et al. [35]	Canada	Cross-sectional	78	Mixed	Mixed	Out-patient	Mixed	11 months	DADDS	8
Emanuel et al. [36]	USA	Cross-sectional	167	Mixed	Mixed	Out-patient	Palliation	3.4 years	DADDS	5
Engelmann et al. [37]	Germany	Cross-sectional	77	Mixed	Advanced	Out-patient	Palliation	4.7 years	DADDS	6
Feng et al. [38]	China	Cross-sectional	586	Gynaecological	Mixed	Not specified	NS	< 1 year	DAS	6
Gonen et al. [39]	Turkey	Cross-sectional	70	Mixed	Mixed	In-patient	Treatment	1.2 years	DAS	6
Gong et al. [40]	China	Cross-sectional	286	Mixed	Mixed	In-patient	NS	NS	DAS	6
Hong et al. [41]	China	Cross-sectional	252	Mixed	Advanced	In-patient	Treatment	NS	DAS (Likert)	5
Kaymak et al. [42]	Turkey	Cross-sectional	40	Mixed	Advanced	In-patient	Palliation	9.2 months	DAS	5
Kiyak and Polat [43]	Turkey	Cross-sectional	140	Breast	Mixed	Out-patient	Treatment	10 months	DAS	5
Krause et al. [44]	Canada	Cross-sectional	60	Mixed	Advanced	Out-patient	NS	2.5 years	DADDS	6
Lau et al. [45]	Hong Kong	Cross sectional	173	Lung	Mixed	Not specified	NS	NS	DAS	5
Liu et al. [46]	China	Cross-sectional	328	Mixed	Advanced	In-patient	Treatment	2.1 years	DADDS	7
Lo et al. [1]	Canada	Cross-sectional	33	Mixed	Advanced	Not specified	NS	2.9 years	DADDS	7
Loughan et al. [47]	USA / Canada	Cross-sectional	105	Brain	Mixed	Out-patient	NS	4.4 years	DDS	8
Loughan et al. [48]	USA / Canada	Cross-sectional	81	Brain	Mixed	Not specified	NS	NS	DAS	6
Loughan et al. [48]	USA / Canada	Cross-sectional	109	Brain	Mixed	Not specified	NS	NS	DADDS	6
Mansori et al. [49]	Iran	Cross-sectional	230	Gynaecological	Mixed	Not specified	Treatment	1.3 years	DAS (Likert)	4
Neel et al. [50]	Canada	Cross-sectional	60	Mixed	Advanced	Out-patient	NS	2.5 years	DADDS	6
Philipp et al. [24]	Germany	Longitudinal	206	Mixed	Advanced	Not specified	NS	3.2 years	DADDS	6
Scheffold et al. [51]	Germany	Cross-sectional	190	Mixed	Advanced	Both	NS	3.3 years	DADDS	7
Shapiro et al. [52]	Canada	Cross-sectional	305	Mixed	Advanced	Out-patient	Treatment	NS	DADDS	6
Sherman et al. [53]	USA	Cross-sectional	38	Mixed	Advanced	Both	Treatment	NS	DAQ	6
Shirkavand et al.[54]	Iran	Cross-sectional	185	Mixed	Advanced	In-patient	Treatment	1.2 years	DAS	6
Soleimani et al. [16]	Iran	Cross-sectional	330	Mixed	Mixed	Both	Treatment	< 1 year	DAS (Likert)	6
Su et al. [55]	China	Cross-sectional	200	Breast	Advanced	Not specified	NS	6.3 years	DADDS	6
Şahan et al. [56]	Turkey	Cross-sectional	60	Mixed	Mixed	Out-patient	Treatment	NS	RDAS	5
Tang et al. [57]	China	Cross-sectional	256	Mixed	Advanced	Both	Treatment	NS	DADDS	5
Tang et al. [58]	Taiwan	Cross-sectional	219	Mixed	Advanced	Not specified	Treatment	NS	DAS (Likert)	8
Turan and Dural [59]	Turkey	Cross-sectional	260	Mixed	Mixed	Out-patient	NS	NS	DAS	5
Uslu-Sahan et al. [60]	Turkey	Cross-sectional	200	Gynaecological	Mixed	In-patient	Treatment	< 6 months	RDAS	5
Yang et al. [61]	China	Cross-sectional	330	Mixed	Mixed	Not specified	NS	NS	DAS (Likert)	8

*DA* death anxiety, *DADDS* death and dying distress scale, *DAS* death anxiety scale, *DAS (Likert)* DAS with 5-point Likert scale from strongly disagree to strongly agree, *DAQ* death anxiety questionnaire, *NS* not specified, *RDAS* revised death anxiety scale

All but two studies examined the effects of death anxiety in adult cancer patients and survivors. Two studies [36, 54] examined death anxiety specifically in older adults (55 +) and no studies investigated the relationship between death anxiety and psychosocial outcomes in young adults, adolescents, or children. Studies were conducted from a range of countries (see Table 1). Twenty-one studies were conducted in collectivist countries including China (*n* = 7), Turkey (*n* = 6), and Iran (*n* = 4). Twenty studies were conducted in individualist countries including the USA (*n* = 6), Canada (*n* = 6), and Australia (*n* = 2).

### Cancer variables

Most studies (71%) recruited mixed cancer samples. Forty-one percent of studies examined death anxiety specifically in people living with advanced cancer. We found no studies which chose to examine death anxiety solely in people with early-stage cancer. Fourteen (34%) studies conducted their research in outpatient settings, eight (20%) in in-patient settings, five (12%) across both settings, and 14 did not specify a setting. Of the studies which reported a duration since diagnosis, the average was 2.6 years (*SD* = 1.77).

### Severity of death anxiety

Overall, participants in the meta-analysis had an average score of 8/15 (*SD* = 2.83) for death anxiety on the Death Anxiety Scale (DAS) [62]. This suggested that death anxiety amongst people living with and beyond cancer was in the moderate range. The severity of death anxiety was moderated by culture. On the DAS, those from individualist cultures had significantly lower death anxiety than individuals from collectivist cultures (*z* = 6.90, *p* < 0.01). On a more recently developed measure, the Death and Dying Distress Scale (DADDS) [1], the mean for the sample was 26/75 (*SD* = 7.77) which also placed people in the moderate range. However, the differences between cultures on this measure were in the opposite direction. That is, those from collectivist cultures had significantly lower death anxiety scores than those from individualist cultures (*z* = 13.37, *p* < 0.01).

### Psychosocial outcomes and moderator analysis

After determining effect sizes for each outcome variable, moderator analyses were conducted where there were a sufficient number of studies. Covariates tested were culture, age, cancer stage, cancer type, cancer setting, and treatment approach. A summary of the findings is presented in Table 2. Other potential moderators, such as religious background, sexuality, or ethnicity of the sample, were not reported in a sufficiently consistent way to allow these variables to be analysed as moderators.

**Table 2:** Summary of findings from meta-analysis

	Effect				Heterogeneity					
	*r*	95% CI	*Z*	*p*	*Q*	df	*p*	*I*^2^	Tau	Tau^2^
FCR (*k* = 8)	0.626	0.555 , 0.687	13.254	0.000	18.686	7	0.009	62.538	0.122	0.015
Anxiety (*k* = 14)	0.586	0.491 , 0.667	9.865	0.000	104.284	13	0.000	87.534	0.235	0.055
Depression (*k* = 18)	0.510	0.431 , 0.581	10.819	0.000	97.559	17	0.000	82.575	0.038	0.195
Demoralisation (*k*= 3)	0.671	0.619 , 0.718	17.739	0.000	2.326	2	0.313	14.009	0.031	0.001
QOL (*k* = 6)	− 0.530	− 0.636 , − 0.403	− 7.123	0.000	23.724	5	0.000	78.924	0.174	0.030
Self-esteem (*k* = 4)	− 0.420	− 0.491 , − 0.342	− 9.699	0.000	3.975	3	0.264	24.536	0.046	0.002
SOM (*k* = 5)	− 0.380	− 0.460 , − 0.294	− 8.060	0.000	9.819	4	0.044	59.264	0.084	0.007

*FCR* fear of cancer recurrence, *QOL* quality of life, *SOM* sense of meaning, *r* correlation, *CI* confidence interval, *df* degrees of freedom

#### Anxiety

Fourteen studies examined the association between death anxiety and anxiety. A significant positive correlation was found between the two variables (*k* = 14, *r* = 0.59, 95%CI [0.49, 0.67], *p* < 0.001), with a large effect size but significant heterogeneity (*Q*_13_ = 104.28, *I*^2^ = 88%, *p* < 0.001). A significant effect of age was found. That is, as age increased, the association between anxiety and death anxiety decreased (*β* =  − 0.02, 95%CI [− 0.03, − 0.002], *p* < 0.05). The results of the meta-regression indicated that age explained 41% of the variance in the association (*R*^2^ = 0.41, *Q*(1) = 4.98, *p* < 0.05). No other covariates moderated the association between anxiety and death anxiety (*p* > 0.05).

#### Depression

Eighteen studies examined the association between death anxiety and depression. A significant positive correlation was found (*k* = 18, *r* = 0.51, 95%CI [0.43, 0.58], *p* < 0.001), with a large effect size and significant heterogeneity (*Q*_17_ = 97.56, *I*^2^ = 83%, *p* < 0.001). A significant effect of culture was found (*Q*(1) = 4.006, *p* = 0.045). That is, for those studies conducted in a collectivist culture, the association between death anxiety and depression was stronger (*r* = 0.59 (95%CI [0.49, 0.67], *p* < 0.001) than those in an individualist culture (*r* = 0.46 (95%CI [0.36, 0.54], *p* < 0.001). No other significant covariates were found (*p* > 0.05).

#### Fear of cancer recurrence

Eight studies measured the association between death anxiety and FCR. A significant positive correlation was found (*k* = 8, *r* = 0.63, 95%CI [0.56, 0.69], *p* < 0.001), with a very large effect size and moderate heterogeneity (*Q*_7_ = 18.69, *I*^2^ = 63%, *p* < 0.01). Moderator analyses were conducted, and no significant covariates were found. Notably, the effect of culture could not be tested as all studies were conducted in western-individualist cultures.

#### Quality of life

Six studies measured the association between QoL and death anxiety. A significant negative correlation was found (*k* = 6, *r* =  − 0.53, 95%CI [− 0.64, − 0.40], *p* < 0.001), with a large effect size and significant heterogeneity (*Q*_5_ = 23.72, *I*^2^ = 79%, *p* < 0.001). Moderator analyses found a significant effect of treatment approach (*Q*(1) = 4.372, *p* = 0.037). Those in palliative care had a significantly greater association between death anxiety and QoL (*r* =  − 0.63 (95%CI [− 0.77, − 0.45], *p* < 0.001) compared to those in active treatment (*r* =  − 0.33 (95%CI [− 0.55, − 0.08], *p* < 0.05). No other significant covariates were found (*p* > 0.05).

#### Demoralisation

Three studies measured the association between death anxiety and demoralisation. A significant positive correlation was found (*r* = 0.67, 95%CI [0.62, 0.72], *p* < 0.001), with a large effect size and no significant heterogeneity (*Q*_2_ = 2.33, *I*^2^ = 14%, *p* = 0.32 > 0.05).

#### Self-esteem

Four studies measured the association between death anxiety and self-esteem. A significant negative correlation was found (*r* =  − 0.42, 95%CI [− 0.49, − 0.34], *p* < 0.001), with a moderate effect size and no significant heterogeneity (*Q*_3_ = 3.98, *I*^2^ = 25%, *p* = 0.26 > 0.05). No significant covariates were found (*p* > 0.05).

#### Sense of meaning

Five studies measured the association between death anxiety and sense of meaning in life. A significant negative correlation was found (*r* =  − 0.38, 95%CI [− 0.46, − 0.29], *p* < 0.001), with a moderate effect size and minimal heterogeneity (*Q*_4_ = 9.82, *I*^2^ = 59%, *p* = 0.044). No significant covariates were found (*p* > 0.05).

### Publication bias

For all analyses, we inspected the funnel plots for symmetry. For all but two outcomes, there was a low likelihood of bias across studies. For self-esteem and sense of meaning, funnel plots indicated a likelihood of negative skew from missing studies. The fail-safe *N* was 136 and 208, respectively, indicating a small likelihood of bias for these two outcomes, although in each case it is likely that the relationship exists, but that the estimates of effect size might be overstated.

### Quality assessment

The quality of included studies was generally good (Table 1). Inclusion criteria, participants, and study setting were usually well defined and described. However, studies often failed to specify who was responsible for data collection, whether they were trained in administering measures, and who was responsible for determining whether a patient had cancer (i.e., oncologists, nurses, or self-report). Many studies did not report on the type of cancer patients were diagnosed with, nor the treatment approach, making it difficult to explore moderator effects. Most studies identified potential confounding variables; however, few studies controlled for these variables. See Supplementary Table 3 for detailed assessment results for each study.

## Discussion

The aim of this review was to quantify the relationship between a range of psychosocial outcomes and death anxiety in people living with or beyond cancer, and to identify moderators of these relationships. Overall, we identified moderate levels of death anxiety, according to the two most commonly used measures of death anxiety. Interestingly, there were cultural differences whereby people from collectivist cultures reported lower death anxiety on DAS but higher death anxiety on the DADDS than people from individualist cultures. Death anxiety was strongly associated with depression, anxiety, FCR, poor QoL and demoralisation. Smaller, but still significant, relationships with death anxiety were identified for self-esteem and sense of meaning, although the latter findings may be overestimated due to publication bias. We found three important moderator results: (1) the younger the person, the stronger the relationship between anxiety and death anxiety; (2) those in palliative care had a stronger relationship between QoL and death anxiety than those currently in active treatment; and (3) those from a collectivist culture had a stronger relationship between depression and death anxiety than those from an individualist culture.

There were large and robust associations between death anxiety and all psychosocial outcomes analysed. Specifically, we found death anxiety was significantly correlated with higher levels of anxiety and depression across studies with large effect sizes. This finding is consistent with the meta-analysis by Li et al. [15], empirical evidence outside the cancer literature [63], and aligns with the transdiagnostic model of death anxiety [9]. According to this model, death anxiety is an underlying factor contributing psychological distress even amongst those who do not have a life-limiting illness. Therefore, it is not surprising that the relationships between psychopathology and death anxiety are also strong in people living with or beyond cancer. Similarly, the very large correlation between death anxiety and FCR highlights the fact that existential issues and FCR are inextricably linked [6, 64].

Our analyses also revealed that the treatment approach moderated the relationship between death anxiety and QoL. It was interesting to find a heightened association between death anxiety and diminished QoL among palliative care patients who are generally closer to the end of their life and it is possible that those who continue to experience death anxiety as their illness progresses are at higher risk of deterioration of quality of life [36]. As improving quality of life is the objective of palliative care [65], factors associated with QoL, such as death anxiety, warrant investigation. Psychologists are too infrequently represented in palliative care settings [66] and these results align with the professional consensus that more psychological resources are needed in the context of palliative care [67]. Our findings suggest palliative care interventions that focus on existential issues, such as death anxiety, are worthy of research since addressing death anxiety particularly for those at the end of their life may enhance overall QoL at the end of life.

A gap identified in our review is the scarcity of studies focusing on early-stage cancer patients and younger populations, including adolescents and young adults. This is important, considering younger cancer patients experience higher rates of death anxiety [15] and FCR [68]. Young cancer patients face unique challenges, including concerns about the future and the social implications of their diagnosis. Despite the absence of studies focusing specifically on young cancer survivors, our results found that death anxiety is more strongly associated with overall anxiety amongst younger people. Hence, the need for research into death anxiety amongst young people should be emphasised.

Our findings provide preliminary evidence for the influence of culture in experiencing death anxiety. We found that people from collectivist cultures exhibited a stronger association between death anxiety and depression compared to those from individualist cultures. There are several reasons why people in collectivist cultures who have high levels of death anxiety may be more prone to depression. Collectivist societies emphasise interconnectedness and communal identity, which may lead to a heightened awareness of death’s implications for the group [20]. Alternatively, in some collective cultures, patients sometimes prefer not to discuss death with their medical team or family [69] and it is possible that this lack of communication also results in a lack of support. While these explanations are speculative, cultural beliefs about death, the afterlife, and individual identity are likely to intersect to shape how one perceives and reacts to their mortality [16].

Interestingly, rates of death anxiety in collectivist or individualist cultures varied depending on the chosen measure. It was found that those from individualist cultures reported higher death anxiety when measured with the DADDS. The DADDS was initially developed and validated in cancer patients from an individualist culture (Canada) [70]. The measure has a two-factor structure encompassing existential regrets and fears of directly experiencing death [70], which may assess more individualist aspects of death anxiety. Thus, our finding may reflect the cultural validity and relevance of the measure for cancer patients from this demographic.

In contrast, those from collectivist cultures scored higher on the DAS. The DAS, developed in 1970, has not been validated in a sample of cancer patients. One item, for example, is particularly unsuited to a cancer population, ‘I am not particularly afraid of getting cancer’. Items from this measure are concerned with the awareness of death, e.g., ‘the thought of death never bothers me’, as opposed to fears associated with the experience of dying. It is known that the DAS appears to have a different factor structure when applied across different cultures [71] and our results suggest that the DAS taps into concerns that are more common in collectivist cultures.

Widely varied factor structures are a common problem across available measures of death anxiety and make the conceptualisation of the construct difficult [72]. For those with cancer, the DADDs measure, which accurately captures fears associated with the experience of dying, may be most clinically relevant to people with cancer [1]. Zuccala et al. [72] in their systematic review of death anxiety measures pointed out the absence of sound psychometric properties amongst existing measures. Our results suggest it is imperative for researchers to consider the validity and cultural relevance of a measure when examining death anxiety in collectivist or individualist cultures.

Yet, generalising cultural norms across nations risks oversimplifying the diverse and often heterogeneous nature of cultures within modern societies [73]. Individuals within the same cultural group may have vastly different beliefs and attitudes towards death, shaped by personal experiences, family dynamics, and local traditions. Understanding these mechanisms requires more than correlational studies; it necessitates qualitative exploration of how cultural narratives and social support systems interact to shape individual experiences of death anxiety.

### Clinical implications

It is clear from previous research that there is a lack of quality intervention trials for death anxiety in cancer patients and survivors [13]. Our study confirms that death anxiety is an important construct for those who are living with or beyond cancer. Overall levels of death anxiety were moderate, and death anxiety was strongly associated with all psychosocial outcomes. Hence, death anxiety is an important target for intervention.

Our results suggest that the experience of death anxiety might differ according to culture, which underscores the importance of developing culturally sensitive approaches to managing and measuring death anxiety. Most of the previous intervention studies have been conducted in Western cultures [13]. Thus, there is a need for further understanding of the nature of death anxiety in collectivist cultures, and adaptation of interventions to better address the nature of fears in different cultures.

## Limitations

The results of any meta-analysis are only as strong as the data from the original studies. Most studies were of good quality, although future research could better address confounders. There are many potential confounders of the role of culture that we identified in this meta-analysis, including likely differences in religiosity, sexuality, or ethnicity. Unfortunately, there was insufficient data in the majority of trials for us to reliably extract this data and compare groups on the basis of those characteristics. Likewise, individual studies that purposefully sampled people from different cultures and measured the degree of collective versus individualistic culture for each individual would be more sensitive to help us understand the differences observed in this meta-analysis.

Similarly, while there were a large number of studies overall, moderator analysis was limited due to a lack of consistent reporting of cancer and medical variables across studies. In some analyses, particularly those examining different cultures on different measures, there were relatively few studies in some comparison groups (e.g., *k* = 3). As such, primary research comparing individuals from collectivist and individualist communities on the two most common measures of death anxiety would be welcome, particularly research that explored reasons for these differences in terms of other likely differences between samples drawn from different cultures. In the meantime, these differences should be interpreted cautiously. Finally, whilst our study considered the role of culture in death anxiety, we were limited by our inclusion of studies published in English. As such, future research should seek to review this effect in studies published in other languages. These limitations notwithstanding, we did identify robust relationships between death anxiety and the outcomes of interest, that did not appear to be due to publication bias and stemmed from studies of reasonable quality. Therefore, we can be confident in the observed relationships. Nevertheless, as we investigated cross-sectional relationships, whether death anxiety is a cause or effect of poor psychosocial functioning cannot be determined.

## Conclusion

Death anxiety is a salient yet complex construct for people living with or beyond cancer. This review identified several preliminary factors which may moderate the experience of death anxiety in this population. Understanding these factors at an individual level is crucial for psychological interventions to adequately address death anxiety and its correlates. An approach that integrates cultural, psychological, and medical perspectives will be essential in improving mental health outcomes for individuals confronting the reality of cancer and mortality.

## Supplementary Information

Below is the link to the electronic supplementary material.

ESM 1 DOCX (28.0KB)

## Data Availability

Data are available upon reasonable request to the corresponding author.
