# Supplementary material for: A meta-analysis of death anxiety in people living with or beyond cancer: the important role of culture
Source: J Cancer Surviv. 2025 Jul 4;20(Suppl 1):752–63. doi: 10.1007/s11764-025-01856-8 (PMC13676632; doi:10.1007/s11764-025-01856-8)
Supplement: Supplementary file 1 [file 11764_2025_1856_MOESM1_ESM.docx]

**Supplementary Material**

A Meta-Analysis of Death Anxiety in People Living with or Beyond Cancer: The important role of culture

Journal of Cancer Survivorship

Audrey Bennett^1^, Louise Sharpe^1^*, Jack B. Boyse^1^

^1^School of Psychology, The University of Sydney, Sydney, NSW, Australia

*Corresponding author: Louise Sharpe, School of Psychology, The University of Sydney, NSW 2006. Louise.sharpe@sydney.edu.au

## **Supplementary Table 3: Quality Assessment Results**

| Author (Year) | Inclusion Criteria | Participants and setting | Objective cancer measurement | Reliable measurement | Confounds identified | Confounds addressed | Valid measures | Appropriate statistical analyses |
| --- | --- | --- | --- | --- | --- | --- | --- | --- |
| Abbas et al.^27^ |  |  |  |  |  |  |  |  |
| Afrashteh & Masoumi^28^ |  |  |  |  |  |  |  |  |
| An et al.^29^ |  |  |  |  |  |  |  |  |
| Berlin & von Blackenburg^30^ |  |  |  |  |  |  |  |  |
| Berlin et al.^31^ |  |  |  |  |  |  |  |  |
| Bibi & Khalid^32^ |  |  |  |  |  |  |  |  |
| Bovero et al.^33^ |  |  |  |  |  |  |  |  |
| Braun et al.^34^ |  |  |  |  |  |  |  |  |
| Coutts-Bain et al.^35^ |  |  |  |  |  |  |  |  |
| Curran et al.^36^ |  |  |  |  |  |  |  |  |
| Eggen et al.^37^ |  |  |  |  |  |  |  |  |
| Emanuel et al.^25^ |  |  |  |  |  |  |  |  |
| Engelmann et al.^38^ |  |  |  |  |  |  |  |  |
| Feng et al.^39^ |  |  |  |  |  |  |  |  |
| Gonen et al.^40^ |  |  |  |  |  |  |  |  |
| Gong et al.^41^ |  |  |  |  |  |  |  |  |
| Hong et al.^42^ |  |  |  |  |  |  |  |  |
| Kaymak et al.^43^ |  |  |  |  |  |  |  |  |
| Kiyak & Polat^44^ |  |  |  |  |  |  |  |  |
| Krause et al.^45^ |  |  |  |  |  |  |  |  |
| Lau et al.^46^ |  |  |  |  |  |  |  |  |
| Liu et al.^47^ |  |  |  |  |  |  |  |  |
| Lo et al.^1^ |  |  |  |  |  |  |  |  |
| Loughan et al.^48^ |  |  |  |  |  |  |  |  |
| Loughan et al.^49^ |  |  |  |  |  |  |  |  |
| Loughan et al.^49^ |  |  |  |  |  |  |  |  |
| Mansori et al.^50^ |  |  |  |  |  |  |  |  |
| Neel et al.^51^ |  |  |  |  |  |  |  |  |
| Philipp et al.^24^ |  |  |  |  |  |  |  |  |
| Scheffold et al.^52^ |  |  |  |  |  |  |  |  |
| Shapiro et al.^53^ |  |  |  |  |  |  |  |  |
| Sherman et al.^54^ |  |  |  |  |  |  |  |  |
| Shirkavand et al.^26^ |  |  |  |  |  |  |  |  |
| Soleimani et al.^16^ |  |  |  |  |  |  |  |  |
| Su et al.^55^ |  |  |  |  |  |  |  |  |
| Şahan et al.^56^ |  |  |  |  |  |  |  |  |
| Tang et al.^57^ |  |  |  |  |  |  |  |  |
| Tang et al.^58^ |  |  |  |  |  |  |  |  |
| Turan & Dural^59^ |  |  |  |  |  |  |  |  |
| Uslu-Sahan et al.^60^ |  |  |  |  |  |  |  |  |
| Yang et al.^61^ |  |  |  |  |  |  |  |  |
